# Editorial: Teleostean forebrain organization and evolution: Links to behavior and ecological niche

**DOI:** 10.3389/fnana.2023.1167451

**Published:** 2023-03-07

**Authors:** Mónica Folgueira, Nobuhiko Miyasaka

**Affiliations:** ^1^Grupo Neurover, Centro Interdisciplinar Bioloxía e Química (CICA), Universidade da Coruña, A Coruña, Spain; ^2^Grupo Neurover, Departamento Bioloxía, Facultade de Ciencias, Universidade da Coruña, A Coruña, Spain; ^3^Laboratory for Systems Molecular Ethology, RIKEN Center for Brain Science, Saitama, Japan

**Keywords:** teleosts, forebrain, homology, telencephalon, neuroanatomy, evolution, brain diversity

Teleosts are the most successful group within the vertebrate lineage in terms of number of extant species and conquest of very diverse habitats. The first teleost appeared at about the middle of the Mesozoic era, becoming the dominant fish type already by the end of this era (Romer, 1959). Teleosts have shown a remarkable capacity of adaptation, being able to live in every possible aquatic environment, even when inhospitable. Extremophile teleosts thrive in very harsh conditions, such as those with very low oxygen or in temperatures below freezing (Plath et al., 2015). We can even find fish species whose embryos survive for long periods in dry mud, as is the case for the annual killifishes of the genus *Nothobranchius* (Terzibasi Tozzini and Cellerino, 2020). However, even for teleosts that are adapted to such diverse habitats, rapid climate change is a major problem that threatens the maintenance of the diversified species.

Adaptations to different ecological niches resulted in a high variety of body morphologies and behaviors in teleosts, as well as sensory and motor specializations that reflects in a diversity of brain morphologies (see Ito et al., 2007). This is, for instance, clearly exemplified with mouth anatomy, feeding strategies and the specialized organization of gustatory centers in the hindbrain (see figure 4 in Ito et al., 2007). As a result of this diversity, the teleost group lends itself particularly well to studies of comparative neuroanatomy and evolution, as evidenced by their long historical use in such studies.

There remains a lot to learn about how genetic and epigenetic factors affect developmental events that lead to changes in brain structure. The tools developed recently as a result of the introduction of zebrafish (*Danio rerio*, Fam. Cyprinidae) as a model system in research will certainly enable advances in our understanding (Wyatt et al., 2015). In the past, most studies analyzing the organization and structure of teleostean brains have been performed in adults. For the very few studies performed during developmental stages, they were largely descriptive and non experimental. This is changing with the expanded focus on zebrafish. A number of new tools are now available to us, including transgenic lines and mutants, making experimentation in developmental stages more feasible. This puts us in a much better position to tackle the mechanisms behind the generation of brain diversity.

The teleostean forebrain has some unique traits compared to other vertebrates. One of these is the presence of an everted telencephalon, which contrasts with the evaginated telencephalon of other vertebrates. Eversion was thought to occur as a simple lateral out-folding of the neural tube during development, however some recent studies have challenged that view (Nieuwenhuys, 2011). Due to the remarkable differences between the morphology of everted and evaginated telencephali, the teleostean telencephalon poses a big challenge for comparisons and establishing homologies with vertebrate brains, especially for the pallium and subpallium. Although some advances have been made (Braford, 2009; Mueller and Wullimann, 2009; Biechl et al., 2017; Mueller, 2022), many questions remain open.

Determining homologies between teleosts and other vertebrates, especially with mammals, is one of the pursuits in the fields of comparative neuroanatomy and evolutionary biology (Nieuwenhuys, 2009). This remains challenging for certain areas, because of the unique traits of the teleostean brain. Recently, this question has been a priority due to the extended use of zebrafish for biomedical research. There is no doubt that establishing homologies is a very important issue in evolutionary science, however finding homologies is also very challenging and requires a complex approach (Striedter and Northcutt, 1991). Even when we focus on common traits in the comparison between brains, we should never forget the differences. The following quote draws our attention to this issue very clearly:

“*The natural emphasis on discovering commonalities, homologies and analogies that simplify has distracted us from the main result of evolution, which is to create differences”—Bullock (**2002**)*.

As scientists we also need to be mindful not to err on the side of generalizing results from one species to a whole group, a trap in which it is very easy to fall as a result of enthusiasm for our own research.

The present Research Topic includes three original research articles, two reviews and an opinion article addressing some of the items discussed above. Teleosts show different degrees of eversion and complexity of the pallium. Demski and Beaver present a very comprehensive description of the cytoarchitecture of the tectal-related pallium of two species of squirrelfish (Fam. Holocentridae, Order Beryciformes), which have a highly specialized visual system that they use to hunt in coral reefs. Turner et al. describe early events in the development of the everted telencephalon of zebrafish, based on the expression of various transgenes and neuroanatomical markers. Data sets were registered to reference brains, generating a resource for the scientific community. Teleosts, along with their sister group holosteans, have been used to study the evolution of neurochemical systems in vertebrates, including the catecholaminergic system (Lozano et al., 2019). Borgonovo et al. describe the distribution of tyrosine hydroxylase (TH)-immunopositive neurons in the brain of turquoise killifish *Nothobranchius furzeri* (Fam. Nothobranchiidae; Order Cyprinodontiformes). This species is a promising model for understanding aging and neurodegeneration due to the fastest maturation and the shortest captive lifespan known in vertebrates. Lal and Kawakami review earlier studies that have dealt with the complex issue of the organization, homologies and function of the vertebrate amygdala. They also review genetic approaches, which have recently become available through development of transgenic zebrafish, for further understanding of this brain area. Whitlock and Palominos review the teleostean olfactory system and discuss how the alteration of habitats as a consequence of climate change might impact this system. Zebrafish is widely used nowadays in biomedical research; the relevance of this species in translational neuroscience depends largely on homologies with humans. Focusing on these species, Gerlai discusses the great complexity of determining biological conservation especially at a behavioral level, and the approaches necessary to tackle this issue.

In summary, the articles included in this Research Topic expand our understanding of the structure, neurochemistry, development and function of the forebrain in teleosts, and provide us with an opportunity to consider several important aspects, including the impact of climate change on the sensory systems and the use of teleosts in translational research. In addition, these articles highlight some of the new tools and techniques that have been developed in zebrafish, which will likely prove useful for research in other fish species. Teleosts have inspired neuroanatomists and evolutionary biologists for over a century and will certainly continue to do so for many years to come.

## Author contributions

MF wrote the first draft of the manuscript. MF and NM edited the manuscript, read, and approved the final version. All authors contributed to the article and approved the submitted version.
